# Retraction Note: Spatio-temporal analysis and simulation of urban ecological resilience in Guangzhou City based on the FLUS model

**DOI:** 10.1038/s41598-024-80077-y

**Published:** 2024-11-19

**Authors:** Zhenjie Liao, Lijuan Zhang

**Affiliations:** School of Management, Guangzhou Huashang College, Guangzhou, China

Retraction of: *Scientific Reports* 10.1038/s41598-023-33342-5, published online 06 May 2023

The Editors have retracted this Article.

An investigation conducted after its publication discovered that it contains material which substantially overlaps with the work of a different set of authors^1^.

The Authors agree with this retraction.
